# Moisture Absorption Behavior and Adhesion Properties of GNP/Epoxy Nanocomposite Adhesives

**DOI:** 10.3390/polym13111850

**Published:** 2021-06-02

**Authors:** Nurziana Kong, Nur Zalikha Khalil, Holger Fricke

**Affiliations:** 1 Department of Mechanical Engineering, College of Engineering, Universiti Malaysia Pahang, Lebuhraya Tun Razak, Kuantan 26300, Pahang, Malaysia; kongnurziana@gmail.com; 2 Fraunhofer Institute for Manufacturing and Advanced Materials (IFAM), Adhesive Bonding Technology and Surfaces, Wiener Strasse 12, 28359 Bremen, Germany; holger.fricke@ifam.fraunhofer.de

**Keywords:** adhesive joining, graphene nanoplatelet, water absorption, mechanical properties

## Abstract

In the current work, an attempt has been made to investigate the effect of Graphene Nanoplatelets (GNP) reinforcement to water absorption behavior and mechanical properties of adhesive bonding with epoxy. Epoxy adhesive with various GNP content (i.e., 0.0~2.0 wt%) was utilized to joint aluminum adherend subjected to various immersion periods (i.e., 0~60 days). Subsequently, the effect of GNP reinforcement on water uptake, water absorption rate and tensile shear strength was investigated. Depending on GNP content, two distinct behaviors in water uptake and moisture absorption rate have been observed; specimens with lower GNP content (0.5~1.0 wt%) have demonstrated increased/retention of water uptake and water absorption rate regardless of immersion period. Meanwhile, at higher GNP content (1.5~2.0 wt%), decreased water uptake and water absorption rate are generally observed. At similar GNP content, regardless of immersion periods, water immersed specimens generally demonstrate higher or retention of shear strength when compared to specimens at 0-day immersion period. These observations suggest that the relation between moisture absorption behavior and mechanical properties of GNP-reinforced adhesive with GNP content are rather complex which might be attributed to the interplay of several possible mechanisms.

## 1. Introduction

Adhesive joining is a relatively new bonding technology in which an adhesive is used to join various type of substrates. When compared to other joining techniques such as mechanical fastening and welding, adhesive bonding delivers significant advantages, such as the prospect of producing low-cost components, high strength-to-weight ratio, flexibility for joining dissimilar materials, uniform stress distribution and relatively lesser processing requirements [1,2,3,4,5]. Such advantages have made adhesive bonding application highly relevant in many industrial fields such as automotive/aeronautics [2,6] constructions [7,8] marine [9,10] and electronics packaging [11,12].

However, polymer adhesive often suffers in terms of poor humidity and moisture resistance, limiting its applicability in wider range of engineering applications. In particular, some polymeric adhesives tend to absorb moisture in humid environment, which consequently leads to lower interfacial adhesion and bonding performance [13,14]. Moisture/humidity absorbed into adhesive may cause plasticization / swelling which subsequently facilitate interfacial crack growth resulting in decreased joining strength [15,16].

To address the aforementioned concern, recently, the usage of nanocomposite adhesives with organic and/or inorganic nanoadditives has been proposed due to several important advantages. From the literature, nanocomposite adhesives have demonstrated good potential in improving humidity resistance of adhesive joining. Nanocomposite adhesives have proven to be able to reduce water uptake and moisture absorption rate by hindering polymer plasticization /swelling, leading to retention of bonding strength [12,17] through the following mechanisms: i) nanoadditives form tortuous path by acting as an obstacle to water diffusion leading to decreased diffusion rate and/or ii) formation of chemical interface between nanoparticle and substrates decrease water diffusion leading to retention of joining performance [12,18].

To date, significant works have been devoted to assess the viability of nanocomposite adhesive to improve humidity resistance and bonding performance of adhesive joining. For instance, Khoramishad et al. [17] has reported that the inclusion of 0.5 wt% of MWCNT and 3.0 wt% SiC-nano reinforcement into epoxy adhesive has resulted in maximum reductions of water uptake up to 50% and 46.4%, respectively, as compared to pristine epoxy. Moreover, for water-immersed specimens with 0.5 wt% MWCNT and 5.0 wt% SiC content, the strength of adhesive joining has shown improvement up to 89.1% and 113.2%, respectively, as compared to neat adhesive counterpart. Similar findings have been reported by Panta et al. [12] while working with GNP/epoxy nanocomposite adhesive, where a decrease in water uptake and water diffusion rate up to 20% are observed when compared to that of pristine adhesive. It was also reported that at 0.5 wt% concentration of GNP, water immersed adhesive joining specimens have shown an improvement up to 143% increment of bonding strength as compared to neat adhesive at similar immersion period.

On the other hand, Starkova et al. [19] has reported that the inclusion of 0.5 wt% GNP concentration into epoxy resin has demonstrated an increment of water uptake up to 61.7% when compared to pristine epoxy. From other work, as reported by Sugiman et al. [20], the use of Portland cement-filled epoxy demonstrated an increase of water uptake up to 84% at 25% volume fraction filler. Similar finding has been reported by Capiel et al. [21] where it was demonstrated that the inclusion of 3.0 wt% bentonite nanoclay into epoxy based matrix has resulted in rise of water uptake and water diffusion of up to 38.6% and 105.7%, respectively.

It appears that the effect of nanocomposite adhesive usage towards humidity resistance and subsequent bonding strength of adhesive joining has demonstrated several distinct trends in which these variations might be influenced by several factors such as dispersion stability of nanostructure in the polymer matrix, type of nanoreinforcement and the optimum content of nanoaaditives. In the present work, an investigation has been carried out to elucidate the effect of GNP inclusion towards humidity resistance and subsequent mechanical performance of joining specimens with GNP/epoxy nanocomposite adhesive. Fracture behavior of the fractured specimens were also analyzed and characterized to assist with a more holistic insight on the effect of GNP reinforcement towards the above mentioned elements. An attempt has also been made to correlate moisture absorption behavior of GNP/epoxy nanocomposite adhesive, joining performance and fracture behavior of joining adherends.

In current work, two parts structural epoxy adhesive with various GNP content has been subjected to several immersion periods to investigate its effect to moisture absorption behavior (i.e., water uptake and moisture absorption rate). Mechanical performance of bulk GNP-reinforced adhesive samples and single lap joint specimens with GNP nanoadhesive subjected to various immersion periods was evaluated by tensile testing. Microstructural analysis of fracture surface is performed to assess the failure mode of adhesive joining.

## 2. Materials and Methods

### 2.1. Materials

**Figure 1:**
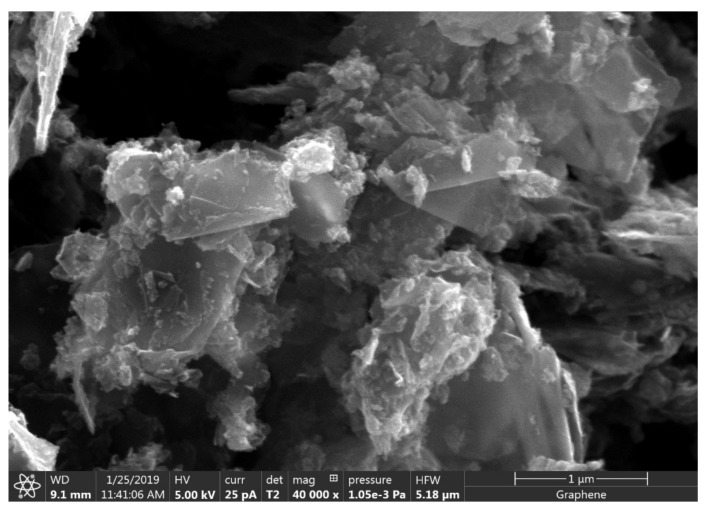
The morphology of as received GNP.

In the present work, a commercially available GNP supplied by Sigma Aldrich (Product Number: 900394, St. Louis, MI, USA) was utilized as the nano-reinforcement (Figure 1). The GNP has a random flake like morphology with the presence of agglomeration. The lateral dimension is observed to be about 1~2 μm which matches the specification provided by manufacturer (<2 μm). A two component structural epoxy adhesive (ALL PURPOSE EPOXY supplied by Republic Chemical Industries Inc, Quezon City, Philippines) with 1:1 weight ratio (viscosity 800,000 cps at 25 °C, cures at 8 h) was used to bond 7075-T6 aluminum alloy adherends. The epoxy resin was based on Epichlorohydrin, Bisphenol A, Aliphatic Glycidylether Modified Bisphenol A and Aliphatic Polyamide Amine. The adhesive is initially without nano-reinforcement where the chemical composition of each part are as follows; part A (<70% Calcium Carbonate as filler, <30% 2,2-bis[p-(2,3-epoxypropoxy) phenyl] propane) and part B (<80% Calcium Carbonate as filler, <20% Polyamide amine).

### 2.2. Specimens and Nanoadhesive Preparation

From former studies of one of the authors [22], it has been demonstrated that a low content of nanoadditives (below 2.0 wt%) has resulted in a positive influence on the mechanical properties of the adhesive without dramatic negative change of physical attributes like viscosity. Following this, in current study, five GNP concentrations were used to prepare nanoadhesive (i.e., 0.0, 0.5, 1.0, 1.5 and 2.0 wt%) to screen this region with positive influence. Three steps of nanoadhesive preparation are involved in this work to improve dispersion of GNP into epoxy matrix. Firstly, the GNP was diluted in ethanol (99.5% absolute denatured C_2_H_5_OH supplied by EMC2 Technology, Selangor, Malaysia) using 1:10 weight ratio mixed using magnetic stirrer (INTLLAB, Shenzen, Guandong, China) for 10 min at 2000 rpm.

**Figure 2:**
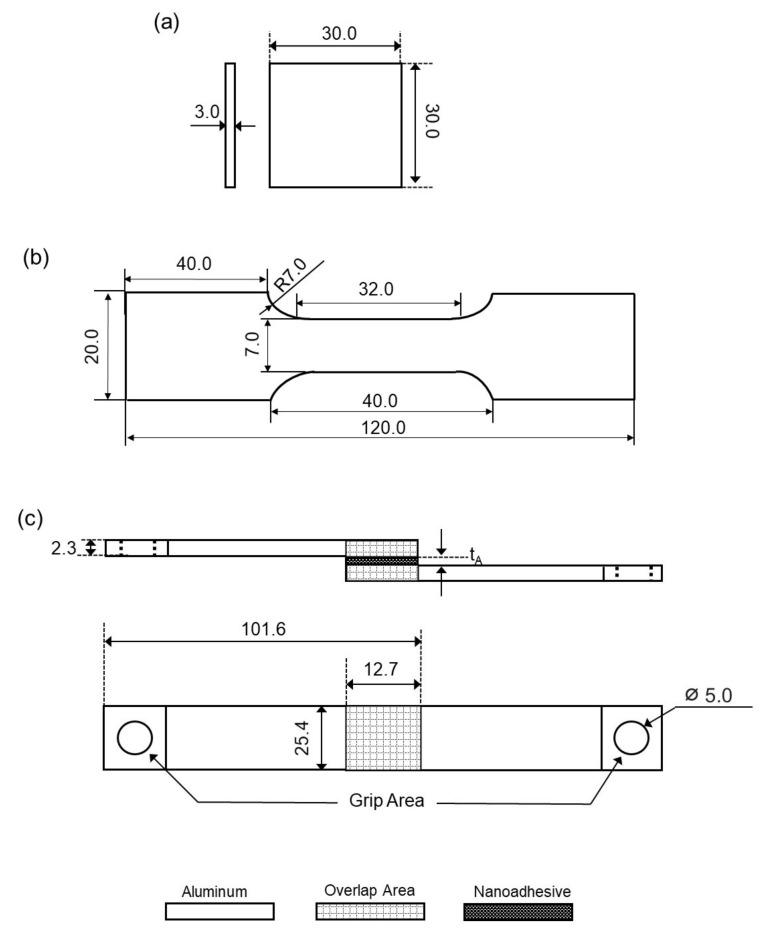
Geometry and dimension of the samples in mm. (**a**) moisture absorption sample, (**b**) tensile test specimen and (**c**) single lap joint specimen.

**Figure 3:**
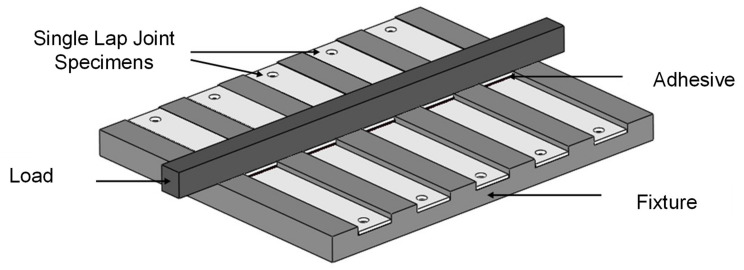
Schematic of fixture used for curing single lap joint specimens.

Subsequently, GNP-ethanol solution was mechanically mixed into part A epoxy using magnetic stirrer (up to 2400 rpm) to improve the dispersion of the GNP into epoxy and was left at room temperature until the ethanol is fully evaporated. It is worth noting that the mixing process was continuously observed to measure the weight difference of the solution in 30 min intervals. The ethanol is considered evaporated when the evaporation rate reaches approximately zero. Subsequently, the solution was mechanically mixed with part B epoxy using 1:1 weight ratio until no visible color difference appeared for about 1 minute. For moisture absorption studies, square samples were prepared by using the industrial silicon mold. Meanwhile, tensile specimens single lap joint specimens for tensile shear test were prepared according to the standard ASTM E8 [23] and ASTM D1002, respectively. At least five samples of each moisture, tensile and single lap joint specimens were prepared for each condition to obtain sufficient reliability of the results. The detailed dimensions of both type of specimens are illustrated in Figure 2. The surface treatment process of 7075-T6 aluminum alloy adherends was conducted by using Abrasive blasting equipment (PanBlast^TM^, Woodlands, Singapore) with aluminum oxide powder grits #80 for 60 seconds to provide surface roughness for allowing sufficient adhesion. Surface roughness of treated adherends was estimated to be approximately 2.4 μm. Adherend surface was preliminary cleansed with air gun to remove existing contaminants and then subjected to blasting process with pressure of 621 kPa for 60 s. Adherends were then degreased in ethanol using digital ultrasonic cleaner (GT Sonic VGT-2000, Guandong, China) for 480 s to eliminate residual contaminants. 1.0 ml drop of nanoadhesive was applied onto surface treated adherend which were then set on a customized jig fixture as shown in Figure 3 and left to fully cure for 24 h at room temperature (25 °C) and pressure. The curing time was extended compared to the technical data sheet for two reasons; firstly, to ensure a complete curing of the material and, secondly, due to practical considerations when planning the laboratory operation. A load of 9.81 N was placed on specimens overlapping area to obtain uniform bondline thickness during the curing process. The bondline thickness, ***t_A_*** is estimated to be approximately 0.8 mm ± 0.03 mm.

### 2.3. Water Absorption Studies

Moisture specimens were initially weighed with analytical digital balance with 0.001 g precision. The specimens were immersed in distilled water and left at room temperature (25 °C) for 10 to 60 days immersion period. A total of 5 samples for each concentration and condition were utilized. The immersion was a stationary process where a closed container was used to immerse the samples. Subsequently, the specimens were mechanically dried using free-lint tissue before the final weight is recorded. Water uptake and normalized water absorption rate of the nanocomposite adhesives were determined by the following expressions, respectively [24].

(1) $$Water\;uptake,M_{t}\;\left(\%\right)=\frac{W_{f}-W_{i}}{W_{i}}\times 100$$

where *M_t_* is the water absorbed at time at specific immersion period, *W_f_* and *W_i_* are sample final weight after respective immersion period and sample initial weight, respectively.

(2) $$Normalized\;Water\;absorption\;rate,R_{w}\left(\%\right)=\frac{V_{t}}{t\;\left(day\right)\times w}$$

where *V_t_*, *t* and *w* are the volume of water in ml at specific immersion period, immersion period in days and sample final weight, respectively.

### 2.4. Tensile Shear Test

**Figure 4:**
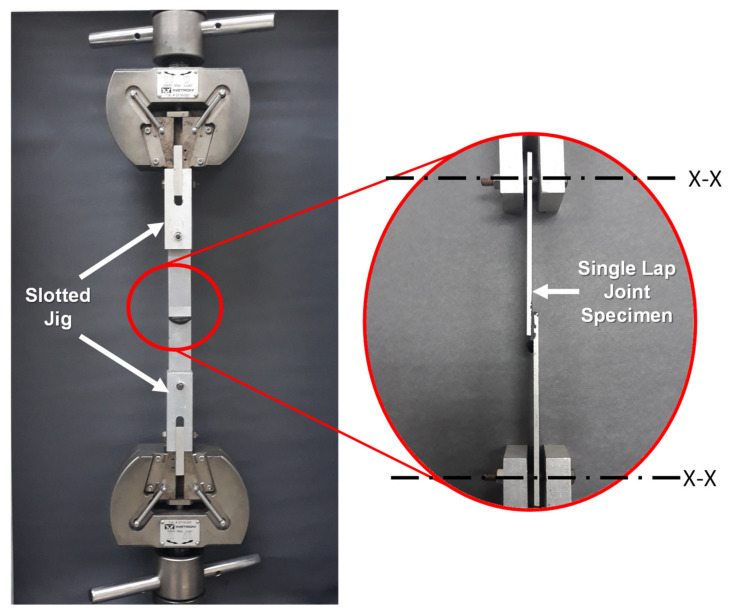
Setup of tensile testing with flexible slotted jig. X-X indicates degree of freedom for the slottedjig.

Five sets of single lap joints specimens of each concentration for each condition were immersed in distilled water at room temperature. Prior to tensile testing, drying process was carried out using free-lint tissue paper followed by drying at room temperature and pressure for 30 min to eliminate surface moisture [25]. The testing was conducted by using Universal tensile machine (INSTRON 3300 Series, Norwood, MA, USA) with 1.3 mm/min crosshead rate and maximum 50 kN load. It is worth noting that tensile testing was conducted by using a customized flexible slotted jig with 1 degree of freedom (1 DOF, 1 translational) along X-X axis to minimize possible occurrence of stress concentration and misalignment in the single lap joint specimen. Figure 4 illustrates the configuration of tensile shear testing.

## 3. Results

### 3.1. Moisture Absorption Behaviour

**Figure 5:**
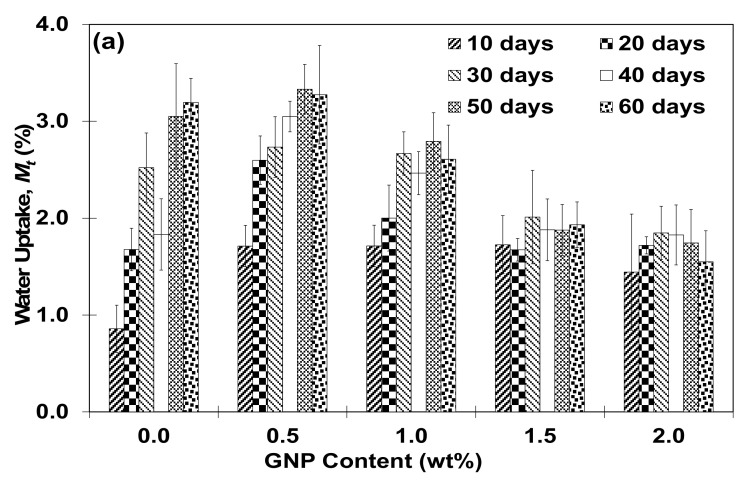

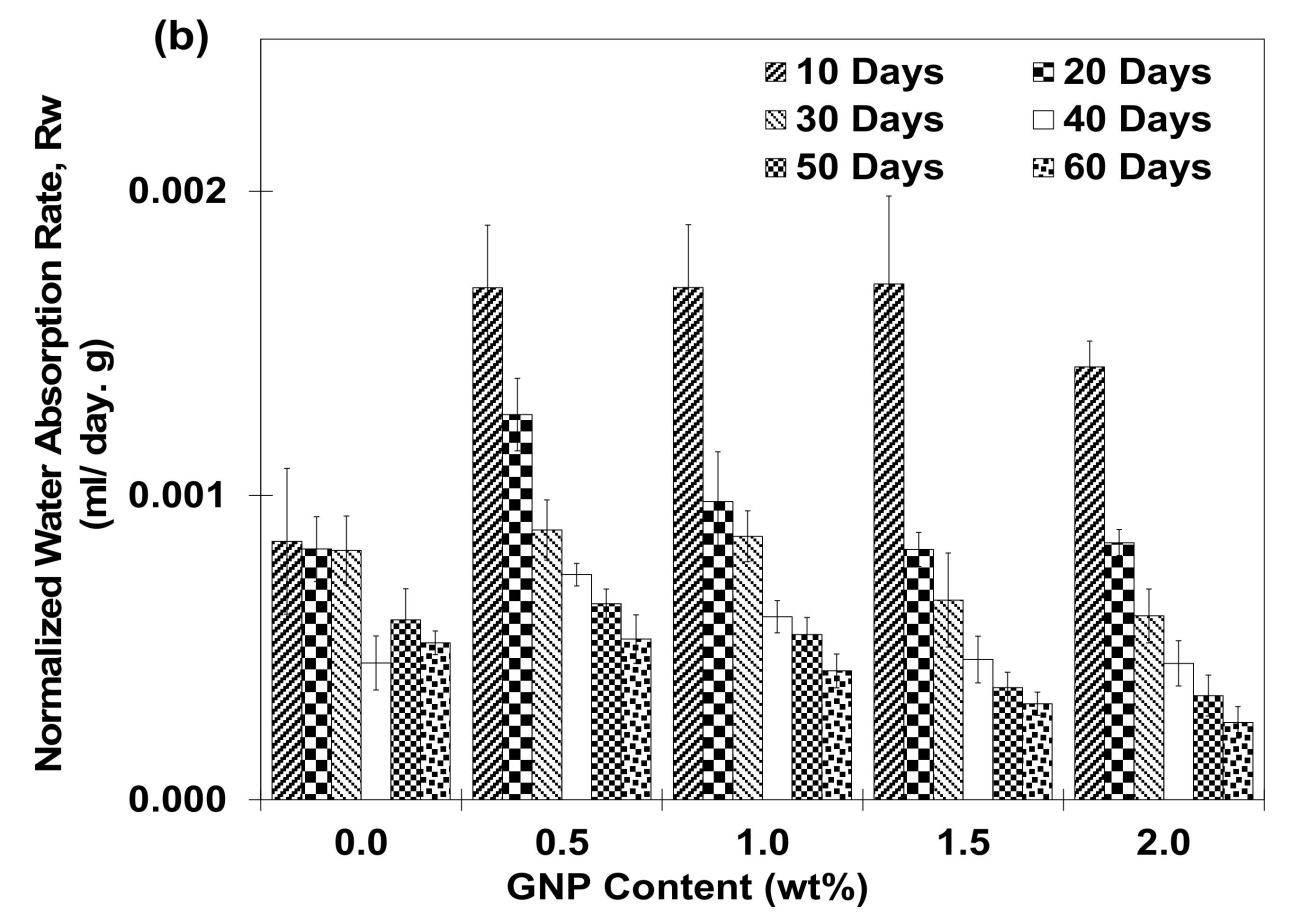
(**a**) Water uptake and (**b**) normalized water absorption rate of GNP-reinforced adhesive as a function of GNP content at various immersion period.

Figure 5 shows the relation between water uptake and normalized water absorption rate as a function of GNP content at various immersion period. From these figures, it can be summarized that when compared to pristine adhesive at similar immersion period, the adoption of GNP into nanoadhesive has generally resulted into several trends in moisture absorption behavior. For water uptake, at lower GNP content (i.e., 0.5 wt% and 1.0 wt%), GNP-reinforced adhesive demonstrated an increased/retention of water uptake regardless of immersion period. However, it appears that there is an exception for 1.0 wt% GNP-reinforced adhesive at 50–60 days, where it recorded lesser water uptake value as compared to pristine adhesive counterpart at similar immersion period. The increment of water uptake in the specimens might be attributed to the insufficient nanostructure content to suppress water uptake into the system, leading to alteration of curing behavior and segments of molecular packing polymer chain when exposed to moist condition. Subsequently, the formation of open polar groups in GNP nanoreinforced epoxy is facilitated resulting in increased hydrophilicity of the system and higher water absorption in the nanoadhesive [12,19,26,27]. Meanwhile, at higher GNP content (i.e., 1.5–2.0 wt%), two trends in water uptake can be observed. At lowest immersion period (i.e., 10 days), nanocomposite adhesive has absorbed higher amount of water as compared to pristine adhesive counterpart at similar immersion period. However, at 20~60 days immersion period, nanocomposite adhesive has demonstrated decreased water uptake. Higher loading of GNP content into the polymer matrix may have facilitated barrier mechanism which have lessen water permeability into nanocomposite adhesive making it more efficient in suppressing water absorption [12]. Similarly, for water absorption rate; (i) at lower GNP content (i.e., 0.5 wt% and 1.0wt%), higher/retention of water absorption rate in the nanoadhesive specimens are observed regardless of immersion period when compared to pristine adhesive at similar immersion period. However, slightly lower water absorption rate is observed for specimens with 0.5 wt% GNP at 60 days immersion period and 1.0 wt% at 50–60 days immersion period (ii) at higher GNP content (i.e., 1.5–2.0 wt%), two trends in water absorption is being observed. At lower immersion period (i.e., 10 and 20 days), higher water absorption rate of specimens is being observed. The higher water absorption rate at lower immersion periods might be due to the filling of water molecules into existing void in the polymer matrix which cause the rearrangement of the small micro-voids resulting in much faster absorption of water molecules into the nanoadhesive [28,29,30]. Meanwhile, at higher immersion period (i.e., 30–60 days), GNP-reinforced adhesive demonstrated lower water absorption rate behavior. As the immersion periods are extended, the voids which had been pre-occupied by water molecules may have resulted in lesser ability of water molecules diffusion resulting in lower water absorption rate. The sufficient amount of nanoreinforcement might have also contributed to lower water absorption rate by permeating lesser amount of water molecules to pass through the epoxy matrix as already mentioned in the introduction section of present article.

### 3.2. Strength and Water Uptake Relation

**Figure 6:**
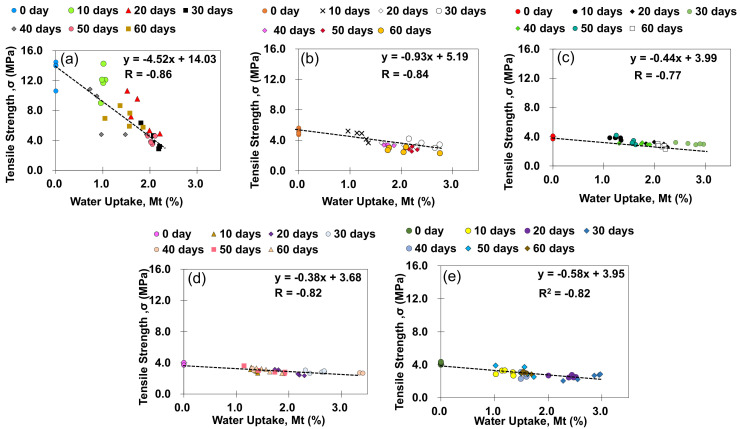
Scatter plot correlation between moisture uptake and tensile strength at various GNP content (**a**) 0.0 wt%, (**b**) 0.5 wt%, (**c**) 1.0 wt%, (**d**) 1.5 wt% and (**e**) 2.0 wt%.

Figure 6 illustrates scatter plot correlation between moisture uptake and strength for tensile bulk specimens at various GNP content and immersion period with its respective trendlines. Linear association between these two variables are also represented by correlation coefficient, *R* [31,32]. It is worth noting that the positive and negative sign in *R* value indicates the tendency of linear relation between the two variables [32]. From the figure, it appears that regardless of GNP content, tensile strength of the specimens shows negatively proportional relation with water uptake, which implies the tendency of strength reduction with increasing moisture content. Moisture absorbed into polymer matrix is often associated with polymer network interactions through secondary bonding mechanisms [33], which may induce strong plasticization and subsequent degradation of the system [34]. Moreover, it is also noted that for tensile specimens with pristine adhesive i.e., 0.0 wt% (see Figure 6a), largest *R* value implies that the strength of tensile specimens is strongly influenced by water uptake of the specimens. On the other hand, as the GNP content increases, it can be observed that the value of *R* is decreasing with the lowest value observed for specimens at 1.0 wt% GNP content which may indicate lesser dependency of strength and moisture uptake in the GNP-reinforced specimens as compared to pristine adhesive counterpart. Possible justification for these observations might be inferred by the barrier properties induced by nanostructure coupled with the stronger formation of chemical interface between polymer matrix and nanostructure thus hindering water progress resulting in retention of mechanical strength [19].

### 3.3. Mechanical Properties

**Figure 7:**
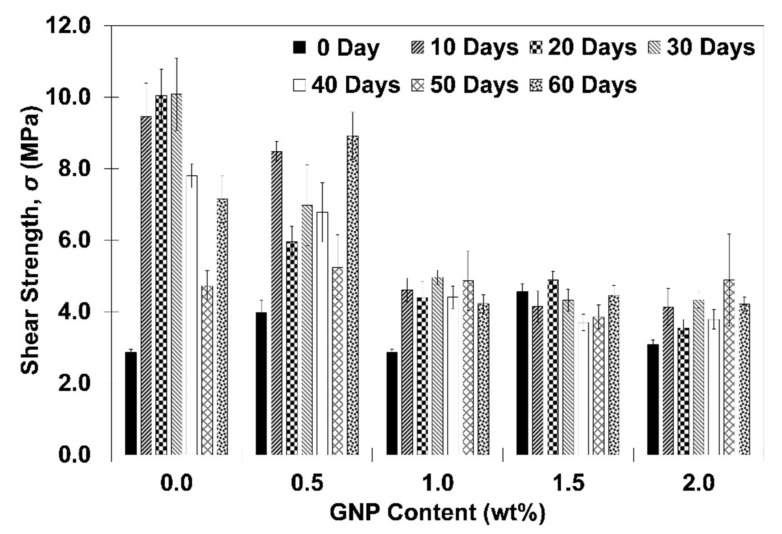
Shear strength of SLJ specimens as a function of GNP content at various immersion period.

Figure 7 shows the shear strength results of adhesive joining with GNP/epoxy nanocomposite adhesive as a function of GNP content and immersion period. In present work, at least five samples of single lap joint specimens were prepared for each GNP content and each immersion period. It can be seen that at dry condition (i.e., 0 day immersion period), pristine adhesive initially demonstrated relatively lower shear strength (i.e., 2.9 MPa ± 0.1 MPa) as compared to the specimens subjected 10~60 days immersion period. It is also worth mentioning that at 0 day immersion period, the inclusion of 0.5, 1.5 and 2.0 wt% GNP content has resulted in some increment of shear strength up to 38.8%, 59.1% and 7.7%, respectively, as compared to neat adhesive counterpart. It appears that for dry specimens, the highest shear strength can be obtained at certain GNP content (i.e., 1.5 wt%) while further inclusion of GNP content has resulted in reduction of bonding performance. This observation is in good agreement with several earlier studies [35,36]. The higher joining strength in specimens with GNP reinforcement than that of pristine adhesive might be attributed to the higher surface to volume ratio of the nanostructure, which results in improved interfacial bonding between polymer matrix and nanostructure facilitating load transfer mechanism [36,37,38]. Meanwhile, it is observed that when compared to pristine adhesive at similar immersion period, specimens with GNP-reinforced adhesive have generally resulted in retention/ deterred bonding performance regardless of GNP content. The decrement in joining performance for water immersed specimens may be associated with plasticization caused by water absorption which weaken the interphase within the GNP and polymer matrix [26,39,40,41] resulting in deterioration of bonding strength. This effect might have been further pronounced by the aggregation of GNP content and the resultant stress concentration leading to crack initiation and propagation [35,42]. Nevertheless, it is also observed that there are few exceptions on specimens with 0.5 wt% GNP at 50~60 immersion period where slight increment in bonding performance is observed. These variations might be associated with the combination of several mechanisms such as the interactions of moisture and polymer matrix and its effect on crosslinking density combined with the chemical barrier like properties by the nanostructure which might have improved the bonding performance as being discussed beforehand [12,18]. Therefore, it can be concluded that within the scope of current work, the effect of GNP inclusion on the shear strength of joining specimens can be concluded as the following; regardless of GNP content, at dry condition (i.e., 0 day immersion period), specimens with GNP inclusion has generally resulted in retention/ improved bonding performance when compared to specimens with pristine adhesive which might be attributed to the higher aspect ratio for GNP [43] thus increasing contact area between GNP with the epoxy matrix, resulting in more efficient load transfer mechanism and improved joining performance [44]. For water immersed specimens, the inclusion of GNP content has generally resulted in deterioration of bonding performance in joining adherend when compared to pristine adhesive counterpart with a few exceptions as discussed above where these variations might be associated with the interplay of several mechanism as discussed beforehand. Nevertheless, with regard to present work, detailed investigation is needed to elucidate the exact mechanism involved.

### 3.4. Fracture Behaviour

**Figure 8:**
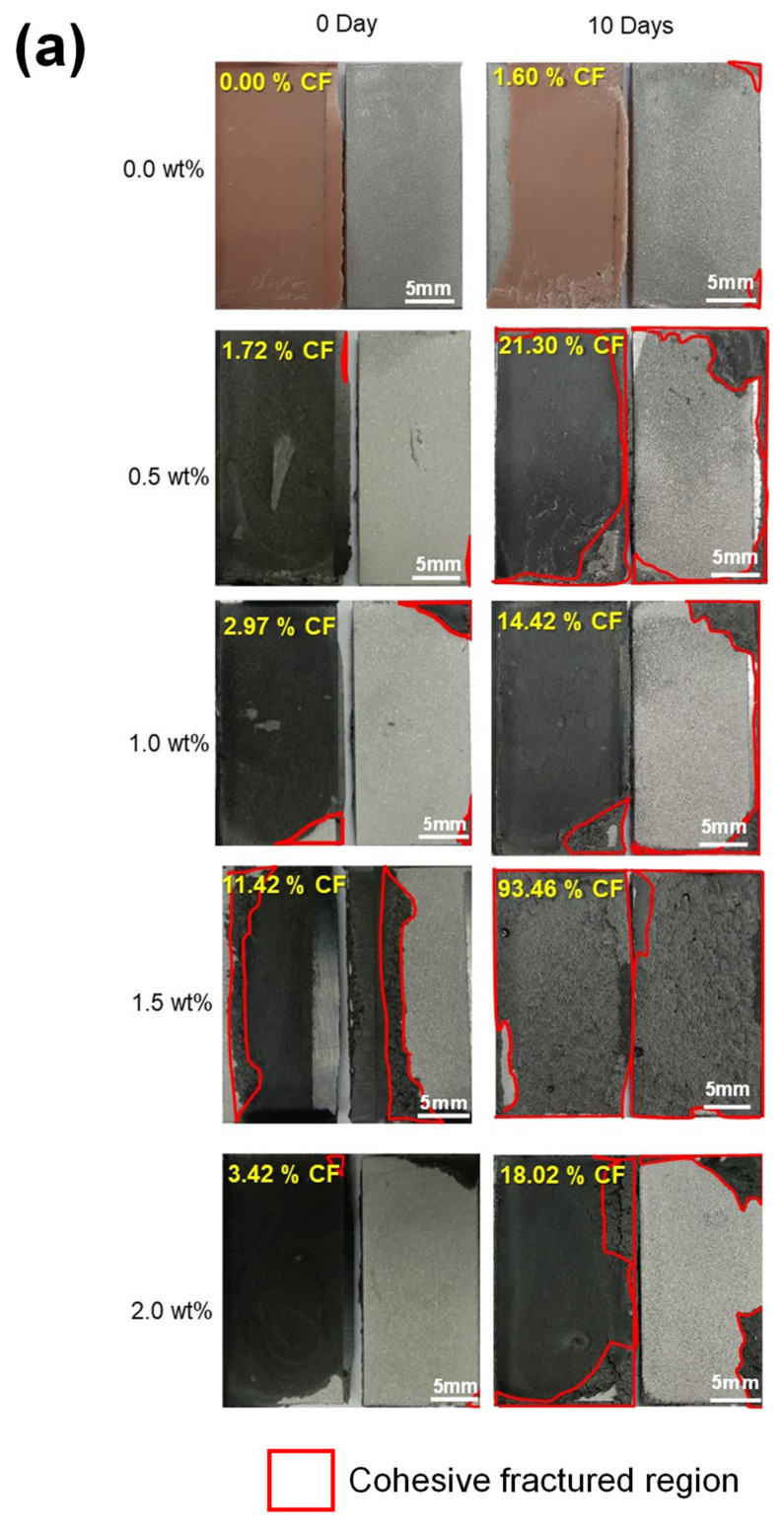

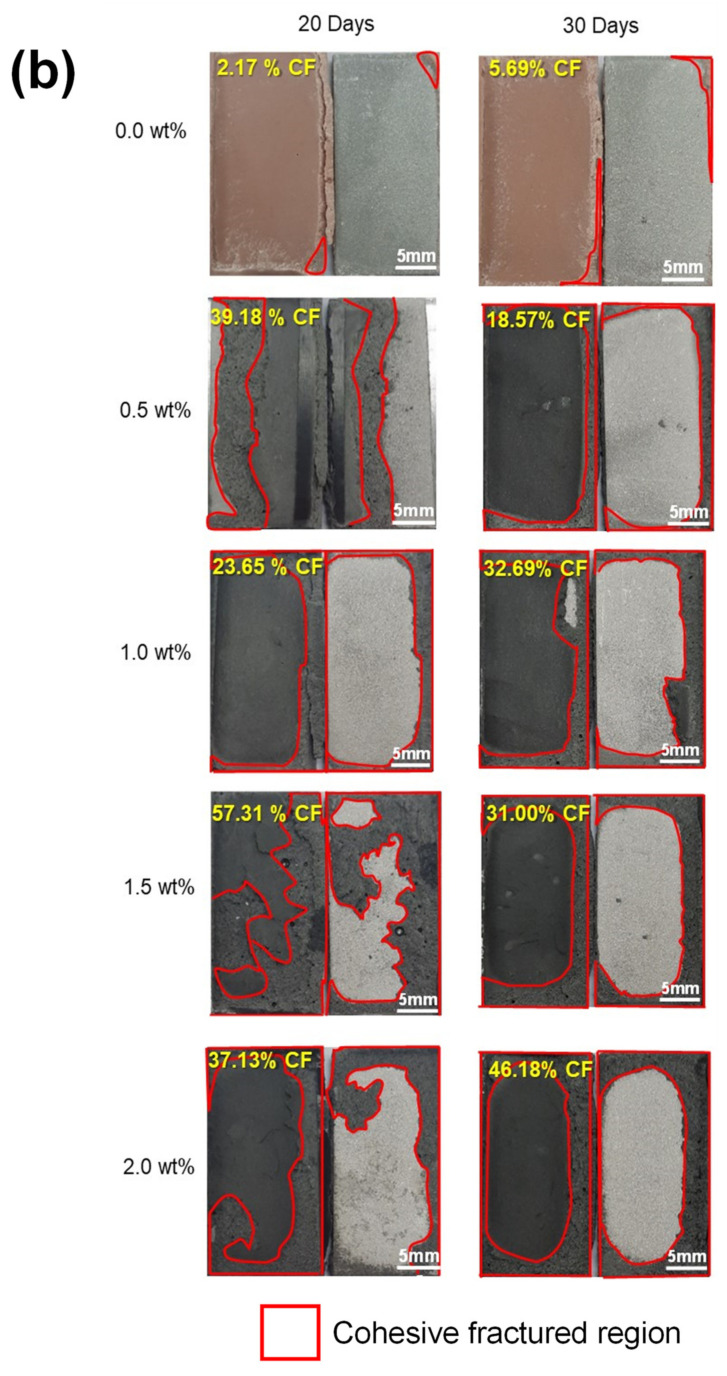

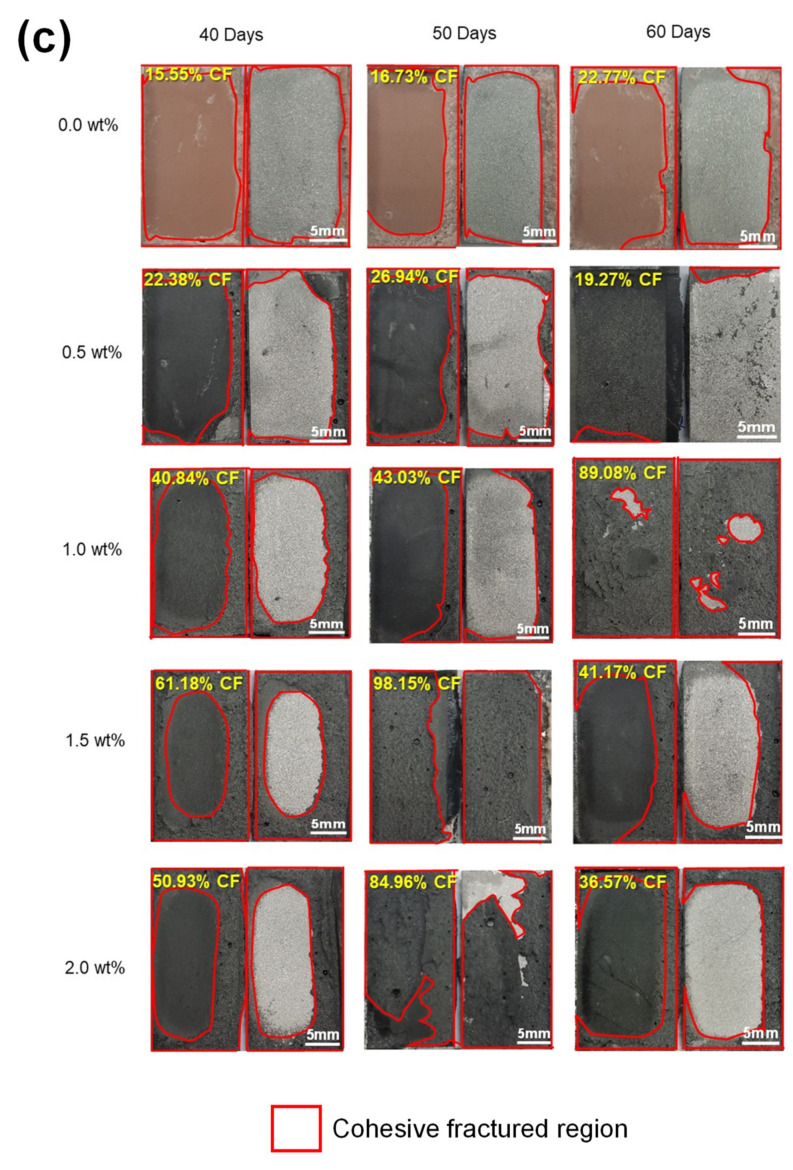
Representative of fracture surface for single lap joint specimens with various GNP concentration at (**a**) 0–10 days, (**b**) 20–30 days, and (**c**) 40–60 days immersion period. Cohesive fractured (CF) mode ratio is shown in the figure.

Figure 8 illustrates the representative images of fracture surface for single lap joint specimens at various GNP concentration and immersion periods. It appears that fractured specimens have demonstrated either adhesive fracture mode (AF), cohesive fracture mode (CF) or the combination of both modes [45]. The CF region is indicated by the red colored lines and the CF fracture mode ratio is also presented in respective images. The identification of AF and CF region was done visually where AF is determined when the fracture occurs between adhesive layer and adherend. AF usually indicates that sufficient adhesion was not achieved. On the other hand, CF is characterized when the failure occur within the adhesive bulk and adhesive is found on both adherends [46]. In many applications, CF is the preferred type of facture since it indicates that sufficient adhesion is obtained and the bond strength is given by the bulk properties of the adhesive [46,47]. In the present work, at least five sets of specimen are utilized for each GNP content and immersion period to obtain an average value. For each of AF and CF mode, the analysis was done by dividing the total surface area for each failure mode with the total area of overlapping area on the joining adherends. In present work, images of fractured specimens were analyzed by using ImageJ open source software version 1.52 where a distinction between the appearance of the failure modes can be made and the percentage of the fracture mode were calculated [22,48].

**Figure 9:**
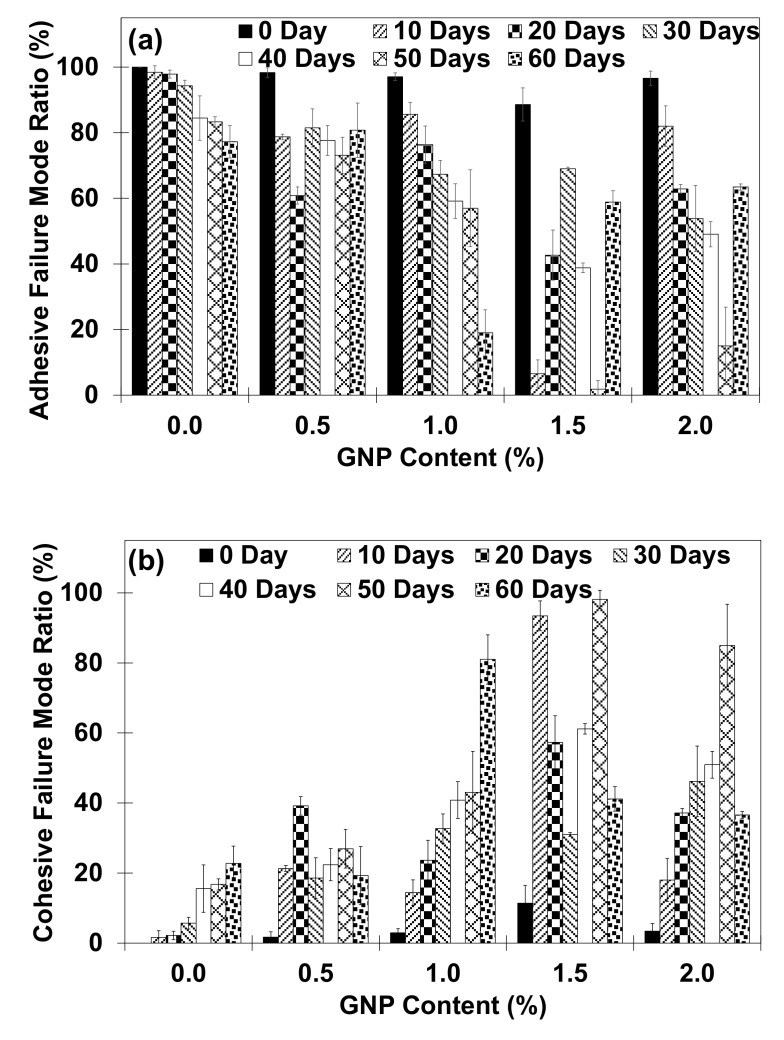
(**a**) Adhesive and (**b**) cohesive failure mode ratio in the single lap joint specimens as a function of GNP content at various immersion period.

Figure 9 summarizes the relation between fracture behavior in the single lap joint specimens at various GNP content and immersion periods.

From these observations, it can be seen that regardless of immersion period, when compared to pristine adhesive counterpart (i.e., 0.0 wt% GNP), GNP-reinforced adhesives have generally demonstrated an increase of CF mode region. Previously, it has been demonstrated that specimens with larger CF region pose higher joining strength generally due to the ductile attribute of the adhesive and adherend interface [49]. The higher CF mode region in the fractured specimens is usually associated with higher energy required to initiate debonding/separation in the joining adherends resulting in higher bonding strength [50,51]. Nevertheless, with regard to present work, the higher CF mode ratio in the joining specimens does not correspond with the higher shear strength of single lap joint specimens as already discussed earlier (see Section 3.3). It is also observed that water immersed specimens have generally demonstrated higher CF mode ratio as compared to the specimens at dry condition (i.e., 0 day immersion period) regardless of GNP content. Moreover, when compared to the pristine adhesive specimens (i.e., 0.0 wt%), specimens with GNP reinforcement have demonstrated a retention/ increased of CF mode region regardless of GNP content at similar immersion period. The higher CF mode region in the specimens with GNP reinforcement may indicate the improved chemical interactions and surface/contact area between GNP and polymer matrix which subsequently improve interphase adhesion between polymer and joining adherend [43,52]. From these results, it is noted that no distinct correlation can be observed between the tensile shear strength and CF mode ratio in the fractured specimens which might be influenced by several factors such as the stability of nanostructure dispersion in polymer matrix and chemical interaction between nanostructure and joining adherend. Therefore, a systematic investigation is needed to elucidate the governing factor which resulted in the abovementioned observations.

## 4. Conclusions

In the present work, an attempt has been made to investigate the effect of GNP reinforcement to the moisture absorption behavior and adhesion properties of adhesive joining with GNP/epoxy nanocomposite adhesive. The following conclusions have been obtained:

1. GNP/epoxy nanocomposite adhesive has demonstrated several moisture absorption and water uptake trends; (i) at lower GNP content (i.e., 0.5 wt% and 1.0 wt%), GNP inclusion has resulted in increased/retention of water uptake and water absorption rate regardless of the immersion period with a few exceptions (ii) at higher GNP content (i.e., 1.5–2.0 wt%), two trends in water uptake and water absorption rate is being observed where at lowest immersion period (i.e., 10–20 days) more water uptake and water absorption rate is observed while lesser water uptake is recorded for specimens at higher immersion period (i.e., 20~60 days).
2. Regardless of GNP content, tensile test result of bulk specimens demonstrated reduction of strength with water uptake increment which is represented by the value of correlation coefficient, ***R***. Smaller ***R*** value in GNP/epoxy nanoadhesive specimens as compared to pristine epoxy imply that the tensile strength of specimens is lesser dependent on water uptake of the specimens where the lowest ***R*** value is observed in specimens with 1.0 wt% GNP.
3. When compared to pristine adhesive specimens, GNP-reinforced nanocomposite adhesive at dry condition (i.e., 0 day immersion period), demonstrate retention/ improved joining strength regardless of GNP content, where the highest shear strength increment of 59.1% is observed at 1.5 wt% GNP inclusion. Meanwhile, when compared to pristine adhesive at similar immersion period, water immersed specimens with GNP nanoreinforcement have generally shown retention/ deterred bonding performance regardless of GNP content.
4. When compared to pristine adhesive counterpart, the inclusion of GNP has resulted in an increase of CF ratio regardless of immersion period. Comparing Figure 7 and Figure 9, the simplest explanation for the decreasing bond strength in the shear tensile tests and the increase in the cohesive fracture pattern seems to be that the addition of GNP weakens the adhesive bulk. Due to a weakening of the bulk by adding GNP, a failure of the bulk occurs at lower forces and correspondingly a higher rate of CF is observed, if the strength of the adhesion remains the same. To clarify this behavior, the strength of bulk samples (i.e., tensile test of the adhesive bulk samples) should be investigated in further tests depending on the addition of GNP.

## Data Availability

The data presented in this study are available on request from the corresponding author.
